# Effect of *Caragana korshinskii* Kom. as a partial substitution for sheep forage on intake, digestibility, growth, carcass features, and the rumen bacterial community

**DOI:** 10.1007/s11250-022-03186-8

**Published:** 2022-05-20

**Authors:** Xiaoqi Wang, Xinyi Huang, Zhichao Zhang, Ziyuan Duan

**Affiliations:** 1 Institute of Genetics and Developmental Biology, Chinese Academy of Sciences, No.1 Beichen West Road, Chaoyang District, Beijing, 100101 China; 2 Lanzhou Institute of Chemical Physics, Chinese Academy of Sciences, Lanzhou, 730000 China

**Keywords:** *Caragana*, Rumen microbiota, Nitrogen utilisation, Volatile fatty acids, Tan sheep

## Abstract

The aim of this study was to verify that *Caragana korshinskii* Kom. (CK) as a component of sheep forage influences lamb digestibility and rumen fermentation by altering the rumen microbial community. Hence, 12 female Tan sheep were allocated into 2 groups: receiving (CK group) or not (control group) 10% of the diet forage fraction with CK. During the 60-day experiment, growth performance, apparent digestibility, rumen volatile fatty acids (VFAs), and nitrogen balance were measured. Meanwhile, the rumen bacterial community diversity and composition were detected by the 16S rRNA sequence. The results indicated that the apparent digestibility of acid detergent fibre (ADF) tended to be higher (0.05 < *P* < 0.10), and the feed conversion efficiency was improved (*P* < 0.05) when CK was offered. Compared to those under *alfalfa*, the composition and abundance of the rumen microbial community were altered in the CK group, and the phylum Firmicutes, which is involved in promoting fibre digestion, increased in abundance. Moreover, VFAs tended to decrease (0.05 < *P* < 0.10), and the molar proportion of butyrate declined; similarly, levels of hypoxanthine and xanthine were lower (*P* < 0.05) in the sheep fed CK and may have been responsible for the decreased abundance of *Fibrobacter* spp., which are cellulolytic ruminal bacteria associated with VFA production.

**Supplementary Information:**

The online version contains supplementary material available at 10.1007/s11250-022-03186-8.

## Introduction

*Caragana korshinskii* Kom. (CK) is widely grown in farming-pasture regions with dry-semidry climates in Northwestern China and is generally used as an afforestation shrub species for artificial vegetation restoration and desertification control owing to its ecological value (Long et al. 2020; Zeng et al. 2017). In contrast to its planting area, documented research on CK is scarce, and only a few papers have focused on this species, with plant-soil feedback, chemical component, and limited genetic studies in the literature. Occasionally, local herders use CK as forage in Northwestern China.

In recent years, CK has begun to receive attention as a forage bush, and it is known to have nutritional ingredients, being rich phenolic acids (gallic acid, syringic acid, tannins, etc.), alkaloids (hypaphorine), protein and mineral elements, and various essential and nonessential amino acids (Long et al. 2020; Luan et al. 2017; Zeng et al. 2017; Zhong et al. 2014). The total phenol content of CK reached 19.02 ± 0.57 mg/g (dry substance) by the alkaline extraction method (Zhong et al. 2014), while that of *Medicago sativa* L. (alfalfa), a commercialised legume with a short life cycle, is lower. Although phenolic acids, especially tannins, were known as antinutritional factors for a long time, studies in recent decades found that the suitable addition of tannins in fodder was beneficial for ruminants (Min et al. 2003). Tannins could reduce rumen nitrogen (N) digestibility, provide antiparasitic activity, and increase the yield of wool and lactoprotein and the ovulation rate in sheep (Min et al. 2003). The above results demonstrate that CK has tremendous potential as a feed forage. However, high amounts of CK (higher polyphenol) supplementation could lead to poor palatability and nutrient antagonism. Several studies reported that dietary polyphenol concentrations (over 2% dry matter, DM) had beneficial effects on sheep (Aerts et al. 1999), but a report indicated no positive relationship between polyphenol intake and growth performance, and there was no statistically significant difference when sheep were fed rosemary leaves as 10% and 20% of their diets (dietary polyphenols were 2000 and 4700 mg/kg DM, respectively) (Moñino et al. 2008). Therefore, CK as a feed additive for sheep should be comprehensively considered with regard to its palatability, nutrition, and the polyphenol contents of the feed.

In addition, rumen microorganisms play a key role in supplying nutrients and energy for ruminants, such as synthetising microbial true protein (MTP) and volatile fatty acids (VFAs) (Henderson et al. 2013), which ultimately are used to generate adipose tissue, the hyoideum, and lactoprotein. As diets with varying nutritional ingredients have an effect on rumen microorganisms (Zebeli et al. 2008), we speculate that supplementing feed with CK may affect N utilisation by influencing the microbial community of sheep.

This study aimed to assess the apparent digestibility, N balance, urinary purine derivatives, and rumen bacteria by partial supplementation of CK in sheep forage to compare nutrient metabolism and rumen bacterial communities between sheep receiving and not receiving CK.

## Materials and methods

### Animals and diets

Tan sheep were selected as the experimental animals, a Chinese indigenous breed belonging to the Mongolian series that is adapted to foraging in northern China. Twelve Tan sheep with similar initial body weight (BW, 27.50 ± 3.33 kg, 3 months of age, females) were purchased from the Ningxia livestock farm. The experimental animals were individually weighed and randomly divided into 2 groups (each with 6 lambs) and then housed in individual metabolic cages (height × length × width, 160 × 120 × 60 mm). The sheep were offered a diet containing 70% grass and 30% concentrate (Table 1 and S1) and fresh water ad libitum for the entire formal experiment. In control, the animals were fed a diet consisting of 70% alfalfa hay and 30% concentrate, and the sheep in the CK group received a diet composed of 60% alfalfa hay, 10% CK hay, and 30% concentrate. The concentrate contained 70% corn and 30% wheat middling and bran (DM basis).

**Table 1:** Ingredients and chemical composition of experimental diets

Item	Control	CK
Ingredients, g/kg DM		
Alfalfa	700.0	600.0
*Caragana korshinskii* Kom	0.0	100.0
Commercial concentrate	300.0	300.0
Chemical composition		
DM, g/kg	903.7	904.4
OM, g/kg DM	883.9	890.3
CP, g/kg DM	154.8	148.9
EE, g/kg DM	19.5	20.0
NDF, g/kg DM	497.0	518.0
ADF, g/kg DM	311.4	326.9
GE, MJ/kg DM	15.8	16.0

*DM*, dry matter; *OM*, organic matter; *CP*, crude protein; *EE*, ether extract; *NDF*, neutral detergent fibre; *ADF*, acid detergent fibre; *GE*, gross energy

Composition (per kg of diet): Na 0.50%; Ca 0.41%; P 0.26%; Cu 11 mg; Zn 32 mg; Fe 37 mg; Mn 32 mg; I 4.90 mg; Se 0.74 mg; Co 0.05 mg

Control, diets without *Caragana korshinskii* Kom. (*n* = 6); CK, diets with *Caragana korshinskii* Kom. (*n* = 6)

All the experimental animals were adapted to the diets for 2 weeks (pre-trial period) and weighed before the 60-day feeding experiment (formal period) (Fig. S1). During both the pre-trial and formal periods, diets were provided twice a day at 7:00 and 17:00 and were offered ad libitum. At the end of the experiment, a 6-day digestibility trial was conducted between day 54 and day 59.

### Sample collection and analysis

The sheep were weighed before the morning feeding at the beginning and the end of the formal period. Offered and refused feed amounts were recorded daily to calculate feed intake. From day 54 to 59, the apparent digestibility of the experimental diets was measured by using metabolic cages equipped with clean trays and tubs for the separate collection of faeces and urine. The diet samples were collected daily and mixed individually. Faeces were collected and weighed daily before the 7:00 feeding. For each sheep, 15% of the total daily excretion was collected in a plastic bag and stored at − 20 °C. Total daily urine was recorded and diluted with a sufficient 50% H_2_SO_4_ solution to keep the pH < 3. Ten millilitres of urine mix solution for each sheep was poured into a plastic tube daily and stored at − 20 °C. On day 59, the faecal or urine samples of each sheep were thoroughly blended, and 10% of the total samples were utilised for the chemical analyses (Zhou et al. 2015). The average daily feed intake (ADFI) was calculated following the removal and weighing of residual feed from the offered feed. Diets and faecal samples were sent to Ningxia Feed Engineering and Technology Research Centre for analysis of DM, organic matter (OM), crude protein (CP), ether extract (EE), neutral detergent fibre (NDF), acid detergent fibre (ADF), and N contents by following the China National or Professional Standards: GB/T 6435–2014, GB/T 6438–2007, GB/T 6432–2018, GB/T 6433–2006, NY/T 1459–2007, and GB/T 20,806–2006, respectively.

At the end of the experiment, on day 60, the ruminal fluid was collected before morning feeding and gathered using oral stomach tubes. Approximately 70 mL of the fluid was collected under a vacuum from each sheep. Digesta samples were measured for pH and immediately strained through four layers of cheesecloth. Afterwards, a 5 mL aliquot was mixed with the same volume of deproteinising solution (100 g metaphosphoric acid and 0.6 g crotonic acid/L) for the measurement of VFAs by using a gas chromatographer (Echrom A90E, Yi Meng Electronic Technology Co., Ltd., Shanghai, China) with a capillary column (AT-FFAP: 30.0 m × 0.32 µm × 0.33 µm, Lanzhou Institute of Chemical Physics, Lanzhou, China) (Zhou et al. 2015). After the 60-day trial, the sheep were slaughtered to determine the carcass characteristics. Warm carcass weight was measured within 30 min. The dressing rate was measured as the percentage ratio of the final body weight at slaughter and the warm carcass weight. The GR value was used to assess the fat content of the lamb carcasses and was measured as the total tissue thickness over the 12th rib 110 mm from the midline with a Vernier calliper.

The urine samples were also analysed for N content by Ningxia Feed Engineering and Technology Research Centre, and the values were used to calculate the N retention ratios. Urinary purine derivatives (PD) were determined using reverse-phase high-performance liquid chromatography (Agilent 1200, Agilent Technologies, Lexington, MA).

Total DNA of the ruminal fluid from each sheep was isolated using the TIANamp stool DNA kit (Tiangen, China). The V3–V4 regions of bacterial 16S rRNA were amplified by using the 341F/805R primer set (Herlemann et al. 2011). The PCR products were sequenced on an Illumina HiSeq 2500 platform (Health Genomics Bioinformatics Technology Co., Ltd., Beijing, China).

### Bioinformatics and statistical analysis

Data of intake, digestibility, growth, ruminal fermentation, and carcass features were analysed by independent-samples *T*-test using SPSS 21 (IBM, Armonk, NY, USA). Differences were considered significant at *P* < 0.05, and trends were significant at 0.05 < *P* < 0.10.

The 16S amplicon sequencing data were quality filtered using FLASH. All sequence analyses were performed in QIIME 1.9.1 according to the QIIME instructions. Further error correction was performed using usearch61 with de novo models, and the remaining sequences were clustered into operational taxonomic units (OTUs) using UCLUST with 97% similarity. Taxonomic assignments of all OTUs were made using the Ribosomal Database Project (RDP) classifier within QIIME and the Greengenes13.8 reference database. Based on OTU tables, diversity indexes were calculated using alpha diversity and rank abundance scripts within the QIIME pipeline, and beta diversity was estimated based on Bray–Curtis distance and displayed by principal coordinate analysis (PCoA). The pheatmap and stats packages of the R4.0.3 were used to normalise and cluster the heatmap of genera (average linkage). Linear discriminant analysis effect size (LEfSe) was used to select the discrepant bacteria and their biological relevance between groups based on the nonparametric factor Kruskal–Wallis rank-sum test, Wilcoxon rank-sum test, and linear discriminant analysis.

## Results

### Growth performance and apparent digestibility

There was no significant difference in the initial and final BW, warm carcass weight, or ADFI between the 2 groups, while the feed-to-gain conversion ratio of the CK group was significantly lower than that of the control (*P* < 0.05). This result indicated that the addition of CK to sheep forage could result in a significantly better feed conversion efficiency (Table 2).

**Table 2:** Effects of diets with or without *Caragana korshinskii* Kom. on the growth performance and carcass traits of Tan sheep

Item	Control	CK	*P* value
Growth performance			
Initial body weight, kg	27.50 ± 3.65	27.50 ± 2.98	0.82
Final body weight, kg	31.90 ± 4.27	32.50 ± 3.06	0.71
Average daily gain, g	73.33 ± 13.15	83.33 ± 11.49	0.61
Average daily feed intake, g/d^§^	988.05 ± 121.30	976.91 ± 112.82	0.73
Feed/gain ratio	13.15 ± 1.61	11.49 ± 1.33	0.03
Carcass measurements^£^			
Carcass weight, kg	16.95 ± 2.48	17.27 ± 1.65	0.82
Dressing rate, %	53.12 ± 3.01	53.22 ± 2.04	0.95
GR, mm^$^	4.29 ± 1.37	4.59 ± 1.56	0.71
Meat pH	6.17 ± 0.31	6.08 ± 0.22	0.62

^§^Average daily feed intake is fresh material weight; ^£^warm carcass; ^$^GR, fat depth over the 12th rib at a point 11 cm from the midline of the carcass

Values are mean ± SD; control, diets without *Caragana korshinskii* Kom. (*n* = 6); CK, diets with *Caragana korshinskii* Kom. (*n* = 6)

Table 3 lists the results of the apparent digestibility and N balance of the Tan sheep fed diets with and without CK. With the exception of EE, the other indices of apparent digestibility of DM, OM, CP, and NDF were higher in the CK group than in the control group (*P* > 0.10), and the digestibility of ADF tended to be significantly higher (*P* < 0.10). Even though the total N intake, faecal N excretion, and N retention were similar in the two groups (both *P* > 0.10), the urinary N excretion tended to be significantly smaller (0.05 < *P* < 0.10), while the ratio of N retention to N intake tended to be significantly higher in the CK group (0.05 < *P* < 0.10).

**Table 3:** Apparent digestibility and nitrogen (N) balance of dietary nutrients in diets offered to sheep with or without *Caragana korshinskii* Kom

Item	Control	CK	*P* value
Apparent digestibility, g/kg			
DM	524.7 ± 32.78	542.2 ± 40.57	0.47
OM	538.4 ± 34.56	553.8 ± 45.53	0.53
CP	675.9 ± 20.64	690.1 ± 31.57	0.22
EE	696.8 ± 31.51	678.0 ± 49.84	0.49
NDF	419.9 ± 56.87	449.5 ± 46.43	0.39
ADF	280.6 ± 46.00	339.9 ± 54.94	0.09
N balance, g/d			
Total N intake	24.32 ± 2.90	23.21 ± 2.73	0.58
Urinary N excretion	10.72 ± 0.98	9.52 ± 0.88	0.09
Faecal N excretion	7.81 ± 1.28	7.24 ± 1.49	0.57
N retention	5.79 ± 1.01	6.44 ± 1.04	0.36
N retention/total N intake, g/g	0.24 ± 0.02	0.28 ± 0.04	0.06

*DM*, dry matter; *OM*, organic matter; *CP*, crude protein; *EE*, ether extract; *NDF*, neutral detergent fibre; *ADF*, acid detergent fibre; values are mean ± SD; control, diets without *Caragana korshinskii* Kom. (*n* = 6); CK, diets with *Caragana korshinskii* Kom. (*n* = 6)

### Rumen fermentation

Whether or not CK was added to the fodder, the ruminal pH was not affected (*P* > 0.10); only the concentration of total VFAs tended to be lower (0.05 < *P* < 0.10) when CK hay was substituted in the feed (Table 4), and no effects were observed for isobranch-chained VFAs between the two groups. Among the VFAs, the molar proportion of acetate was increased and butyrate was decreased significantly in the CK group (*P* < 0.05). Even though the difference in the proportion of propionate did not reach statistical significance, the ratio of acetate to propionate was increased significantly with the addition of CK hay (*P* < 0.05).

**Table 4:** Ruminal pH and volatile fatty acid (VFAs) production in Tan sheep offered diets with or without *Caragana korshinskii* Kom

Item	Control	CK	*P* value
pH	6.68 ± 0.07	6.73 ± 0.08	0.27
Total VFA, mmol/L	71.02 ± 13.94	55.32 ± 9.98	0.08
VFAs, mol/100 mol			
Acetate	65.39 ± 0.72	68.78 ± 1.65	0.01
Propionate	16.83 ± 1.19	15.35 ± 1.21	0.43
Butyrate	13.57 ± 1.36	10.99 ± 1.50	0.02
Iso branched-chain VFAs	3.14 ± 0.76	3.81 ± 0.36	0.25
Acetate/propionate	3.89 ± 0.30	4.53 ± 0.45	0.04

Values are mean ± SD; *n* = 6 per group; control, diets without *Caragana korshinskii* Kom.; CK, diets with *Caragana korshinskii* Kom

### Urinary purine derivatives (PD) and PD nitrogen index (PNI)

The urinary PDs of the experimental sheep consisted mainly of allantoin (approximately 85%) and traces of uric acid, hypoxanthine, and xanthine (13–16% combined). From Table 5, urinary PDs excretion of hypoxanthine and xanthine were significantly lower in the diet containing CK (*P* < 0.05), whereas fractions of allantoin and hypoxanthine tended to be higher (0.05 < *P* < 0.10). Seemingly, PNI was similar for each group, and the total PD was 15.35 mmol/d in the control group versus 13.49 mmol/d in the CK group. The difference between the two groups was obvious but not significant.

**Table 5:** Urinary purine derivatives (PDs) and PD nitrogen index (PNI) in Tan sheep offered diets with or without *Caragana korshinskii* Kom

Item	Control	CK	*P* value
Urinary purine derivative excretion, mmol/d			
Allantoin	12.88 ± 1.36	11.82 ± 2.19	0.38
Uric acid	1.02 ± 0.28	0.80 ± 0.30	0.58
Hypoxanthine	1.04 ± 0.14	0.56 ± 0.03	0.01
Xanthine	0.41 ± 0.05	0.27 ± 0.01	0.03
Total PD	15.35 ± 1.58	13.49 ± 3.09	0.16
PNI	0.068 ± 0.01	0.060 ± 0.01	0.35
Fractions of urinary PD, %			
Allantoin	83.58 ± 0.95	86.12 ± 1.43	0.09
Uric acid	6.64 ± 1.30	6.55 ± 1.48	0.96
Hypoxanthine	6.81 ± 1.10	4.17 ± 0.69	0.09
Xanthine	2.65 ± 0.52	2.04 ± 0.31	0.50

Values are mean ± SD; *n* = 6 per group; control, diets without *Caragana korshinskii* Kom.; CK, diets with *Caragana korshinskii* Kom

### Rumen bacterial community

**Fig. 1:**
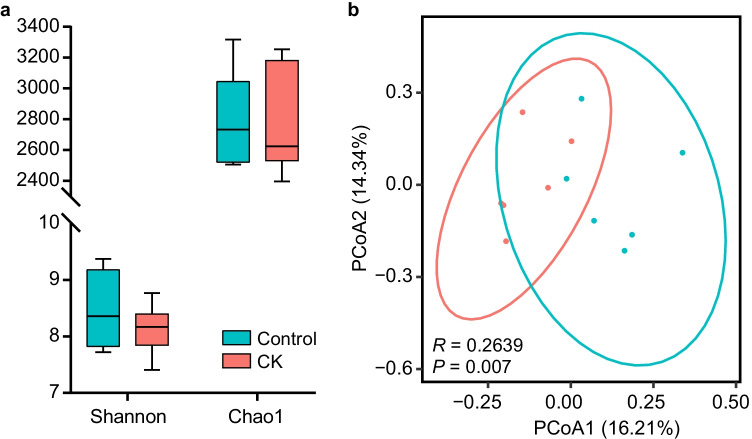
Main rumen bacteria of Tan sheep offered diets with or without *Caragana korshinskii* Kom. **a** Bacterial alpha diversity based on Shannon diversity and Chao 1 indexes. **b** PCoA plot based on the Bray–Curtis dissimilarity of the microbiota. Each dot represents the composition of the microbiota of each sample. Samples were grouped by colour as the labels show. Control: diets without *Caragana korshinskii* Kom., *n* = 6; CK: diets with *Caragana korshinskii* Kom., *n* = 6

**Fig. 2:**
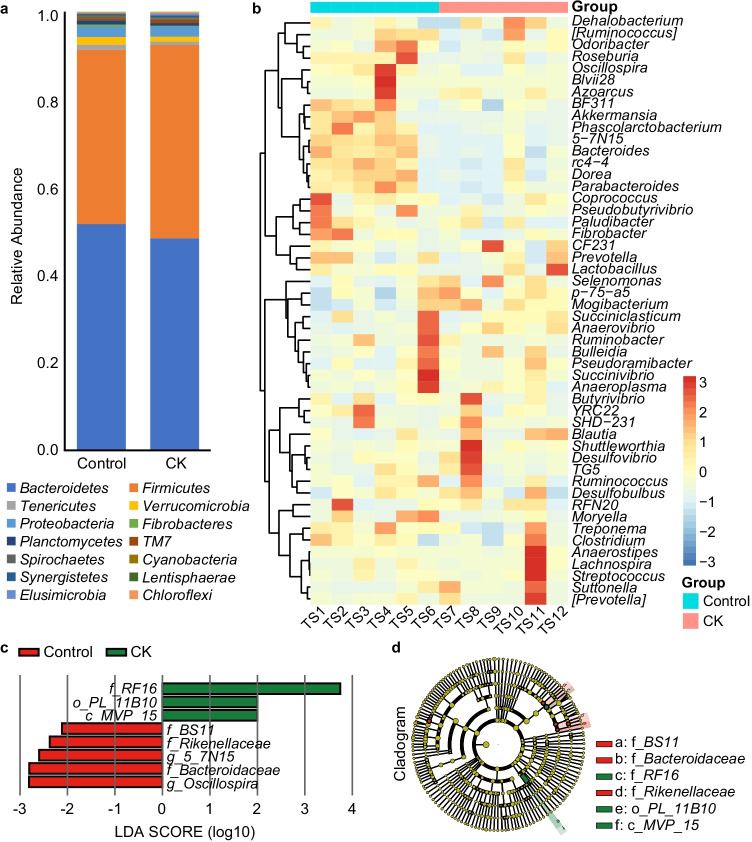
Comparisons of the rumen bacteria relative abundance in Tan sheep offered diets with or without *Caragana korshinskii* Kom. at different classification levels. **a** Bacterial taxonomic composition at the phylum level. The phyla Bacteroidetes and Firmicutes dominated the core microbiome in Tan sheep, followed by the phylum Proteobacteria. **b** A heatmap was generated from hierarchical clustering analysis of the normalised relative abundances of the top 50 genera in the 2 Tan sheep groups. Genera with square brackets are proposed by the Greengenes curators and indicate the recommended taxonomy. **c** Rumen bacteria with LDA scores greater than 2 were speculated to have different abundances between control and CK groups. **d** The cladogram displays the evolutionary relationship at three levels of the taxonomy (class, order, family) for taxa with scores over 2. Control: diets without *Caragana korshinskii* Kom., *n* = 6; CK: diets with *Caragana korshinskii* Kom., *n* = 6

The alpha diversities of the microbiota between the control and treatment groups based on the Shannon diversity and Chao1 indexes are presented in Fig. 1a, with no significant differences. Samples displayed distinct clustering between the 2 groups in Fig. 1b, plotted by the Bray–Curtis distance (*R* = 0.2639, *P* = 0.007). According to 16S RNA sequencing, the dominant phyla in the rumen of the sheep either fed a diet with or without CK were Bacteroidetes, Firmicutes, and Proteobacteria at the phylum level, which accounted for 94% of the total bacterial relative abundance (Fig. 2a). When supplemented with CK, the composition of the bacterial community was altered, and the relative abundances of these three phyla accounted for 48.3%, 44.0%, and 2.3% in the treatment group and 51.6%, 39.8%, and 2.4% in the control, respectively. Although the average relative abundance of the phylum Bacteroidetes decreased 3.3% and Firmicutes increased 4.2% compared to the control group (*P* > 0.10), the difference was non-significant owing to individual variation. However, Verrucomicrobia (*P* = 0.039) and Fibrobacteres (*P* = 0.086) tended to significantly decrease in abundance compared with that in the control group. The abundance distribution of the top 50 genera between the 2 groups is displayed in a genus abundance heatmap, which revealed several genera that exhibited variations between groups (Fig. 2b). Based on the similarity within groups, it was apparent that the bacterial genera *Akkermansia*, *Phascolarctobacterium*, *5-7N15*, *Dorea*, *Bacteroides*, *rc4-4*, *Parabacteroides*, *BF311*, *Coprococcus*, and *Prevotella* were more abundant in the ruminal microbiota of control group, while *Mogibacterium*, *p-75-a5*, and *Selenomonas* were more abundant in that of CK group.

The result of LEfSe showed that 5 and 3 taxa were detected as biomarkers (|LDA|> 2) in the control and treatment groups, respectively (Fig. 2c). Among these eight taxa with significant variation, family BS11, Rikenellaceae, Bacteroidaceae, genus *5_7N15*, and *Oscillospira* decreased, while class MVP_15, order PL_11B10, and family RF16 increased under CK supplementation. Evolutionary relationship analysis showed that families BS11, Rikenellaceae, Bacteroidaceae, and RF16 belong to the order Bacteroidales, which had the highest relative abundance at the order level in both the control and treatment groups, and *5_7N15* was the main genus, with an approximately 50% abundance, in the family Bacteroidaceae. The decrease in abundance in family BS11, Rikenellaceae, and Bacteroidaceae was consistent with the tendency of the phylum Bacteroidetes to decrease in abundance, but the increase in *RF16* was different. PL_11B10 was detected in only one control sample and in five treatment samples and is the unique order of class MVP_15 (Fig. 2d).

## Discussion

Generally, *Caragana korshinskii* Kom. (CK) represented a higher ecological potential than *Medicago sativa* L. (alfalfa) for soil organic carbon, total nitrogen, and phosphorus sequestration in the soil–plant system (Fu et al. 2010), which resulted in widely growing in dry-semidry areas. Meanwhile, due to the strong regeneration ability, CK could be stumped at all seasons. Thus, CK partially replaces alfalfa in planting and sheep feeding would be appropriate from the standpoints of preserving the ecological environment and keeping the balanced nutrition. However, high levels of polyphenol in CK could result in poor palatability and finally affected feed intake, thus 10% of the diet forage fraction with CK hay was a maximum amount under ad libitum in our previous attempts. Most of the few pieces of research showed different forms and states of CK fed sheep, for instance, CK and concentrate were processed into pellet feeds would be conducive to CK intake up to 40% for sheep (Zhang et al. 2021). Or, 10% fermented CK in diet fed sheep could improve the intramuscular fat content (Xu et al. 2020). Furthermore, there is little report on the growth performance of other livestock, such as cattle, with feeding CK.

The two diets (without or with CK) were isonutrient formulated, the main nutrient components, such as OM, CP, EE, NDF, ADF, and gross energy (GE), were considered to be approximately equal in the two groups (Table 1). The experimental data for feeding, digestion, and metabolism showed an increase in the apparent digestibility of ADF, N retention to total N intake ratio, and a decrease in the feed conversion ratio. Less urinary N excretion implied that N utilisation efficiency of Tan sheep was improved with CK supplementation. These results indicated that partial substitution of alfalfa with CK did not lead to any adverse effects but had beneficial results in sheep feeding and digestion, although the contents of NDF and ADF were higher in CK than in alfalfa (Table S1). A study confirmed that when CK was used as an ingredient in the diets of sheep, the degradation rates of DM, NDF, ADF, and CP were up to 70%, 30%, 40%, and 80% at 24 h, respectively (Jiang et al. 2020). The results were consistent with the knowledge that CK is rich primarily in phenolic acids (Zeng et al. 2017), which are natural protein protectants to avoid rapid degradation by rumen microorganisms (Min et al. 2003).

Different to monogastric animals, rumen microbial true protein (MTP) is the main source of amino acids in ruminants (Zhou et al. 2016). Since MTP was proportional to purine derivative (PD) excretion in urine, urinary PD could be used as a practical indicator of MTP synthesis in sheep (Chen et al. 1995). A previous study showed that polyphenols could reduce the concentration of MTP by suppressing rumen bacterial enzyme activity, and this could be determined through the evaluation of urinary PDs (Jones et al. 1994). From our study, although no significant difference was observed between the two groups in total PD excretion, the total PD showed a decreasing tendency, and hypoxanthine and xanthine were lower in the CK supplement group (*P* < 0.05), accounting only for very small amounts of 6–9% combined, which might be in line with previous results. Previous research demonstrated that quebracho tannins had no influence on urinary PD excretion in sheep (Komolong et al. 2001). In any case, our results showed that PD excretion by sheep was closer to that of buffalo than to that previously reported in cattle (Cutrignelli et al. 2007). The PD nitrogen index (PNI) is another indicator that can estimate the efficiency of rumen degradable N conversion to MTP (Makkar and Chen 2004), and neither CK nor polyphenol affected the PNI. Studies have shown that plant polyphenols could inhibit xanthine oxidase in vitro, oxidising hypoxanthine and xanthine (Sabahi et al. 2018), which might have been the principal reason for hypoxanthine and xanthine reduction under CK supply in our study.

In this study, although the results showed that CK had no effect on alpha diversity, richness and evenness of the rumen microbiota, the samples grouped into 2 clusters significantly (*P* = 0.007) according to the Bray–Curtis distance metric (PCoA plot). Hence, feeding CK has a considerable influence on the microbiota composition of sheep. Many reports have proven that rumen microorganisms could be influenced by diet because different bacteria prefer different nutritional ingredients and rumen environments (Zebeli et al. 2008). Consistent with a previous study (Huang et al. 2017), the Bacteroidetes and Firmicutes phyla dominated the core microbiome in both groups of sheep; notwithstanding, the relative abundance of Proteobacteria in sheep that live in a hot environment may increase to become the third core phylum (Qian et al. 2017). Many bacteria in the phylum Firmicutes have the ability to decompose cellulose, and the higher relative abundance of Firmicutes in the CK group could promote fibre digestion and then improve the apparent digestibility of NDF and ADF because of its dominance in the rumen microbiota. Regarding other existing phyla that differed between the two groups, Verrucomicrobia was identified in 1997 and one of the bacterial phyla with aerobic methanotrophic capabilities. The reduction in Verrucomicrobia may have been a consequence of polyphenol changes affected by CK, similar to a study in which polyphenols were supplied in the diets of heifers (De Nardi et al. 2016). The phylum Fibrobacteres was reported to be critical for fibre degradation, and its members could be reduced by polyphenols (Bae et al. 1993), which was also in accordance with our results. Although both of these phyla affected the capacity for fibre degradation via polyphenols, their relative abundances were much lower than that of the high abundance phylum Firmicutes, and a few declining effects were counteracted.

In contrast to the higher relative abundance of the phylum Firmicutes, the genus *Oscillospira* (belonging to the phylum Firmicutes) was decreased in the CK group, and *Oscillospira* is a member of the family Ruminococcaceae, which includes many fibre-degrading bacteria and could be inhibited by polyphenols (McSweeney et al. 2001). An “out of sync” trend also appeared between the phylum Bacteroidetes and its family member *RF16*. Because of low abundance, the significant difference at the family level did not lead to the same difference trend at the phylum level. Moreover, there were the same growth rate trends between the phylum Bacteroidetes and its family members BS11, Bacteroidaceae, and Rikenellaceae, which can utilise a variety of substrates (Solden et al. 2017; Vibart et al. 2019).

To our knowledge, there have been no nutrition studies on the genus *PL_11B10*, as the unique order of class MVP_15 belonging to the phylum Spirochaetes, which includes many cellulolytic microbes that utilise a variety of substrates, such as cellulose, pectin and protein (Hess et al. 2011). According to environmental reports, the genus *PL_11B10* could degrade organic matter in low-salinity petroleum reservoirs. On these grounds, CK as a feed additive may promote *PL_11B10* growth to increase organisms with degradation abilities and aid host growth and metabolism.

According to a previous study (Zhong et al. 2014), the dietary polyphenol levels were approximately 1900 mg/kg (DM) in the CK group, which was similar to those achieved with polyphenol supplementation in sheep (Moñino et al. 2008). Some studies indicated that plant polyphenols could depress sheep or goat rumen fermentation by interacting with the bacterial cell wall or affecting ruminal microorganism enzymes ( Costa et al. 2018) and could be the most effective against cellulolytic bacteria by inhibiting *Fibrobacter* spp. (McSweeney et al. 2001), which belongs to the phylum Fibrobacteres. These microbes have been predicted to produce VFAs in the rumen (Hackmann et al. 2017). Therefore, a decrease in propionate and butyrate, as well as VFAs in the CK group, would be reasonable because of the inhibition of *Fibrobacter* spp. via the polyphenol-rich CK significantly decreased along with a decrease in the abundance of the phylum to which *Fibrobacter* belongs. Additionally, the content of NDF in CK was much higher than that in alfalfa, which was negatively correlated with VFA generation in vitro and in vivo (Njokweni et al. 2019), resulting in a reduction in VFAs in the CK group. The abundances of the VFA producers *Akkermansia*, *Phascolarctobacterium*, *Dorea*, *Bacteroides*, *rc4-4*, *Parabacteroides*, *BF311*, *Coprococcus*, and *Prevotella* (Bi et al. 2018; Feng et al. 2020; O’Hara et al. 2018) were reduced in the ruminal fluid under CK supplementation, still hinting that the reduction in VFAs was influenced by CK through decreasing VFA-producing bacteria.

For ruminants, VFAs provide energy for microbial fermentation and MTP synthesis, while propionic acid can provide more energy than acetic acid (Ben Shabat et al. 2016). Thus, the acetate to propionate ratio could influence the population structure of rumen microorganisms, affecting the nutrient and energy metabolism of the host (Ben Shabat et al. 2016; Sasson et al. 2017). Generally, a lower ratio of acetate to propionate is accompanied by an increase in N deposition for steers and sheep (Foiklang et al. 2016). Conversely, in our study, the higher acetate to propionate ratio of Tan sheep with CK supplementation than without CK was positively associated with N retention. These findings are difficult to explain with previous results and warrant further studies.

## Conclusion

The current study indicated that partial (10%) replaced *Medicago sativa* L. (alfalfa) by *Caragana korshinskii* Kom*.* (CK) in diets seemed to have no influence on gaining weight but remarkable increase in feed conversion efficiency in sheep. Meanwhile, CK could increase the ADF degradation rate, N retention to total N intake ratio, and influence protein synthesis and VFA production through altered rumen bacterial structure, and highly increased the abundance of the phylum Firmicutes. This study preliminarily laid a foundation for using CK as a forage resource for ruminants and further researching CK polyphenol extracts as a feed additive.

## Supplementary Information

Below is the link to the electronic supplementary material.

Supplementary file1 (DOCX 189 KB)

## Data Availability

The datasets used and/or analysed during the current study are available from the corresponding author on reasonable request.
