# Supplementary material for: Effect of Caragana korshinskii Kom. as a partial substitution for sheep forage on intake, digestibility, growth, carcass features, and the rumen bacterial community
Source: Trop Anim Health Prod. 2022 May 20;54(3):190. doi: 10.1007/s11250-022-03186-8 (PMC9123053; doi:10.1007/s11250-022-03186-8)
Supplement: Supplementary file 1 — Supplementary file1 (DOCX 189 KB) [file 11250_2022_3186_MOESM1_ESM.docx]

**Fig. S1** Experimental schedule.


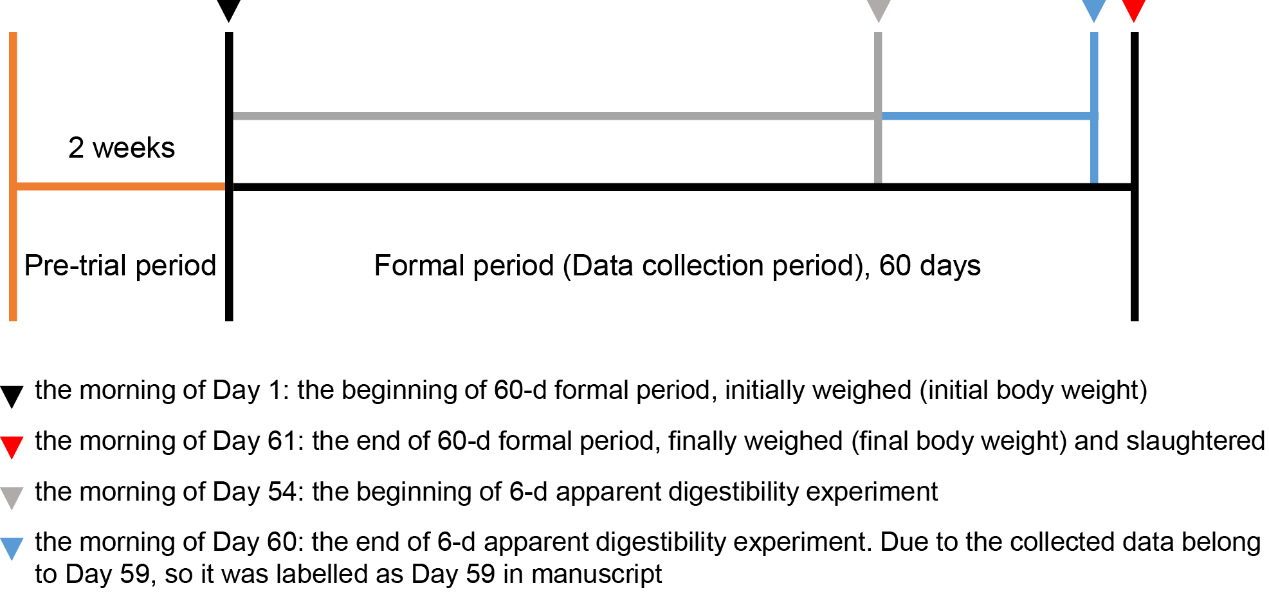


**Table S1** Chemical composition of *Caragana korshinskii* Kom. (CK) and *alfalfa*.

| Item | CK | *Alfalfa* |
| --- | --- | --- |
| Chemical composition, % of DM |  | |
| CP^§^ | 7.99 | 13.22 |
| EE^§^ | 1.56 | 1.14 |
| ADF^§^ | 53.09 | 37.95 |
| NDF^§^ | 67.08 | 46.65 |
| GE, MJ/kg DM^§^ | 18.40 | 15.74 |
| Phenols, mg/g DM^£^ | 19.02 ^11^ | 0.5 ^7^ |

^§^CP, crude protein; EE, crude fat; ADF, acid detergent fiber; NDF, neutral detergent fiber; GE, Gross energy. Chemical composition was tested by Ningxia Feed Engineering and Technology Research Center following China National or Professional Standards: GB/T 6435-2014, GB/T 6438-2007, GB/T 6432-2018, GB/T 6433-2006, NY/T 1459-2007, respectively, and GE was tested by Microcomputer Automatic Calorimeter (ZDHW-5, Wanhe coal testing equipment Co., Ltd, Hebi, China)

^£^Phenol’s content was calculated according to the published references.
